# Phloretin as a Potent Natural TLR2/1 Inhibitor Suppresses TLR2-Induced Inflammation

**DOI:** 10.3390/nu10070868

**Published:** 2018-07-05

**Authors:** Jieun Kim, Prasannavenkatesh Durai, Dasom Jeon, In Duk Jung, Seung Jun Lee, Yeong-Min Park, Yangmee Kim

**Affiliations:** 1 Department of Bioscience and Biotechnology, Konkuk University, Seoul 05029, Korea; za3524@konkuk.ac.kr (J.K.); prasannavenkatesh1@gmail.com (P.D.); dasom921012@konkuk.ac.kr (D.J.); 2 Department of Immunology, School of Medicine, Konkuk University, Chungju 380-701, Korea; jungid@kku.ac.kr (I.D.J.); hahacom@naver.com (S.J.L.); immun3023@kku.ac.kr (Y.-M.P.)

**Keywords:** phloretin, dietary flavonoid, inflammation, TLR2, TLR1, inhibitor

## Abstract

Toll-like receptor 2 (TLR2) responses are involved in various inflammatory immune disorders. Phloretin is a naturally occurring dietary flavonoid that is abundant in fruit. Here, we investigated whether the anti-inflammatory activity of phloretin is mediated through TLR2 pathways, and whether phloretin acts as an inhibitor of TLR2/1 heterodimerization using the TLR2/1 agonist Pam_3_CSK_4_. We tested the effects of phloretin on tumor necrosis factor (TNF)-α production induced by various TLRs using known TLR-specific agonists. Phloretin significantly inhibited Pam_3_CSK_4_-induced TRL2/1 signaling in Raw264.7 cells compared to TLR signaling induced by the other agonists tested. Therefore, we further tested the effects of phloretin in human embryonic kidney (HEK) 293-hTLR2 cells induced by Pam_3_CSK_4_, and confirmed that phloretin has comparable inhibition of TLR2/1 heterodimerization to that induced by the known TLR2 inhibitor CU-CPT22. Moreover, phloretin reduced the secretion of the inflammatory cytokines TNF-α and interleukin (IL)-8 in Pam_3_CSK_4_-induced HEK293-hTLR2 cells, whereas it did not significantly reduce these cytokines under Pam_2_CSK_4_-induced activation. Western blot results showed that phloretin significantly suppressed Pam_3_CSK_4_-induced TLR2 and NF-κB p65 expression. The molecular interactions between phloretin and TLR2 were investigated using bio-layer interferometry and in silico docking. Phloretin bound to TLR2 with micromolar binding affinity, and we proposed a binding model of phloretin at the TLR2–TLR1 interface. Overall, we confirmed that phloretin inhibits the heterodimerization of TLR2/1, highlighting TLR2 signaling as a therapeutic target for treating TLR2-mediated inflammatory immune diseases.

## 1. Introduction

Toll-like receptors (TLRs) are innate immune receptors that defend hosts from microbial infection by detecting common molecular structures in pathogens [1,2]. Among the ten human TLR subfamily members, TLR2 recognizes its ligands in association with TLR1 or TLR6; these ligands are mainly gram-positive bacterial molecules such as lipopeptides, lipoteichoic acids, and peptidoglycans [3,4]. TLR2 is expressed in sentinel immune cells, including dendritic cells, monocytes, and macrophages [5,6]. TLR2 consists of a ligand-binding ectodomain, transmembrane domain, and a Toll/interleukin (IL)-1 receptor domain that initiates the downstream signaling cascade [1,7,8]. TLR signaling then activates nuclear factor (NF)-κB and activating protein-1 (AP-1), which both further induce proinflammatory cytokines [1,9,10]. Phosphorylation of the inhibitor of NF-κB (IκB) allows NF-κB to translocate from the cytoplasm to the nucleus [1,2,10]. Several mechanisms have been identified in the negative regulation of TLR signaling for the control of potentially destructive inflammatory responses [11]. If all of these regulatory mechanisms fail, hyperactivation of TLR2 can cause autoimmune inflammatory diseases such as atopic dermatitis, acne vulgaris, ischemia, and rheumatoid arthritis (RA) [11,12]; thus, TLR2 inhibitors are essential for the control or treatment of these disorders [4,13]. Accordingly, targeting the TLR2-mediated inflammatory response is an important therapeutic target in several immune system-related disorders. Therefore, there is a need to continue to search for naturally derived compounds that can effectively suppress TLR2-activated pathways and to elucidate the underlying mechanisms of this inhibition toward developing new safe and effective drugs for attenuating an excessive inflammatory response.

**Figure 1:**
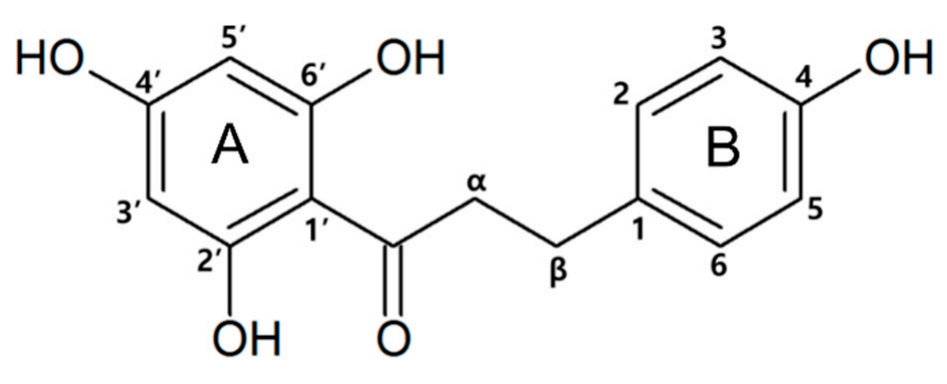
Chemical structure of phloretin.

The natural flavonoid phloretin (3-(4-hydroxyphenyl)-1-(2,4,6-trihydroxyphenyl)-1-propanone) (Figure 1) is a dihydrochalcone, which has A and B two rings with flexible linkage and is abundant in the fruit peel and healthy leaves of the apple (*Malus pumila*) in its free and glucosidic forms: phloridzin and phloretin 2′-*O*-glucose, respectively [14]. Phloretin is a dietary flavonoid with various bioactive properties, including antioxidant effects [15], protection of the skin from ultraviolet light-induced damage [16], anticancer activity [17], antibacterial activity [18,19], and prevention of cardiovascular disease [20]. In addition to these activities, phloretin has been shown to suppress the production of inflammatory mediators such as cytokines, chemokines, and differentiation factors induced by leukocytes, which are stimulated during the innate immune response [21]. TLRs are expressed by cells within the joints of patients with RA, and TLR2 activation was reported to induce migrational and invasive mechanisms, which are critically involved in the pathogenesis of RA [22,23]. Furthermore, phloretin was shown to reduce the damage in a collagen-induced arthritis mouse model by inhibiting several proinflammatory cytokines [24]. However, the inhibitory mechanism of phloretin remains unknown. Since TLR2 is a potential therapeutic target for the treatment of various kinds of inflammatory diseases, the detailed mechanism and involvement of TLR2 in the effects of phloretin on such inflammation suppression should be elucidated. Therefore, in the present study, we investigated whether the inhibitory effect of phloretin is mediated via TLR2 signaling through preventing the heterodimerization of TLR2/1 or TLR2/6.

To develop a potent inhibitor targeting TLRs, it is important to first investigate the specificity and potency. In particular, we aimed to identify the mechanisms involved in the activity of phloretin in TLR2-mediated inflammation. Toward this end, we examined the effect of phloretin on the production of the inflammatory factor tumor necrosis factor (TNF)-α in mouse Raw264.7 cells treated with known TLR-specific agonists. TLR2 recognizes its agonists through heterodimerization with TLR1 or TLR6. For example, the TLR2/1 agonist Pam_3_CSK_4_ activates TLR2/1 heterodimerization, thereby inducing the signaling pathway to activate NF-κB [25]. Pam_3_CSK_4_ is a synthetic lipopeptide with three lipid chains, and is analogous to the acylated amino terminus of bacterial lipoprotein, which is the site of the triggering activity [25]. Similarly, Pam_2_CSK_4_ is a synthetic diacylated lipopeptide that activates TLR2/6 heterodimerization, resulting in a TLR2-mediated signaling cascade [26]. To further investigate whether phloretin is a potent TLR2/1 antagonist in human cells, we explored whether phloretin could inhibit TLR2 signaling by blocking the Pam_3_CSK_4_-induced TLR2/1 dimerization in human embryonic kidney cells transfected with a vector expressing human TLR2 (HEK293-hTLR2 cells). These results were then applied to a docking analysis to propose a binding model for the phloretin–TLR2/1 interaction. This study can help to reveal phloretin as an inhibitor of TLR2/1-mediated inflammatory responses and highlight specific targets for drug development in the treatment and prevention of inflammatory diseases.

## 2. Materials and Methods

### 2.1. Culture of Raw264.7 Cells and HEK293-hTLR2 Cells

HEK293 cells overexpressing and deficient in TLR2 (HEK293-hTLR2 and HEK293-null cells, respectively) were purchased from Invitrogen (San Diego, CA, USA), which were cultured in Dulbecco’s modified Eagle’s medium supplemented with 10% fetal bovine serum, 1% penicillin, 50 µg/mL blasticidin, and 100 µg/mL normocin at 37 °C in a humidified 5% CO_2_ atmosphere. HEK293-hTLR2/6 cells were seeded on poly-l-lysine-coated plates. Mouse macrophage-derived Raw264.7 cells were cultured in Roswell Park Memorial Institute (RPMI) 1640 medium supplemented with 10% fetal bovine serum and 1% penicillin at 37 °C in a humidified 5% CO_2_ atmosphere [27].

### 2.2. Specificity of Phloretin Against Various TLRs

There are several TLRs present in mouse Raw264.7 cells, which have very similar ligand-binding domains to those of human TLRs. Therefore, we tested the effects of phloretin (Sigma-Aldrich, St. Louis, MO, USA) on the TNF-α production induced by various TLRs, including TLR2/1, TLR2/6, TLR3, TLR4, TLR7, and TLR9, using known TLR-specific ligands such as Pam_3_CSK_4_ (200 ng/mL; Invivogen, San Diego, CA, USA), Pam_2_CSK_4_ (200 ng/mL; Invivogen, San Diego, CA, USA), Polyinosinic:polycytidylic acid (Poly(I:C)) (1 µg/mL; Invivogen, San Diego, CA, USA), lipopolysaccharide (LPS; 20 ng/mL; Invivogen, San Diego, CA, USA), imiquimod (1 µg/mL; Sigma-Aldrich, St. Louis, MO, USA), and ODN1826 (10 µg/mL; Invivogen, San Diego, CA, USA), respectively, to selectively activate a particular TLR-mediated TNF-α response. The purity of phloretin was determined to be 99% using high-performance liquid chromatography and mass spectrometry (KBSI, Ochang, Korea). Phloretin was dissolved in dimethyl sulfoxide, and a 10 mg/mL stock solution was used for all experiments. The TNF-α secretion level was measured by enzyme-linked immunosorbent assay (ELISA; R&D systems, Inc., Minneapolis, MN, USA) as previously described [28]. Raw264.7 cells were pretreated with 10 µM and 20 µM of CU-CPT22 (MERK, Darmstadt, Germany) or phloretin for 1 h, and then treated with the respective agonists. After 16 h, the cell culture supernatant was added to precoated plates and incubated for 2 h at room temperature. After washing the plates, the detection antibody (0.4 µg/mL) was added to the plates and incubated for a further 1 h 30 min. All values are expressed as the means ± standard deviations of at least three independent experiments.

### 2.3. Measurement of Inflammatory Cytokines in Pam_3_CSK_4_- and Pam_2_CSK_4_-Stimulated HEK293-hTLR2 Cells

HEK293-hTLR2 and HEK293-null cells were treated with Pam_3_CSK_4_ (100 ng/mL; Invivogen, San Diego, CA, USA) or Pam_2_CSK_4_ (100 ng/mL; Invivogen, San Diego, CA, USA). HEK293-hTLR2 and HEK293-null cells were pretreated with 10 µM and 20 µM of CU-CPT22 and phloretin for 1 h, and then treated with 200 ng/mL of Pam_3_CSK_4_ or Pam_2_CSK_4_. After 16 h, the culture supernatant was collected and TNF-α and IL-8 secretion was measured with an ELISA kit. The detection antibody (0.4 µg/mL) of IL-8 was added to the plates and incubated for a further 1 h 30 min. All values are expressed as the mean ± standard deviation of at least three independent experiments. The range of hTNF-α detection was 15.6–1000 pg/mL and the limit of human interleukin (hIL)-8 detection was 31.2–2000 pg/mL. The range of mean coefficient of variation (CV%) was similar for singleplex ELISAs at 4.3–23%.

### 2.4. Cytotoxicity of Phloretin Against Mouse Raw264.7 and Human HEK293-hTLR2 Cells

The cytotoxic effects of phloretin and CU-CPT22 on cell survival were evaluated in HEK293-hTLR2 cells using the 3-(4,5-dimethylthiazol-2-yl)-2,5-diphenyltetrazolium bromide (MTT; Sigma-Aldrich, St. Louis, MO, USA) assay, which measures the cellular metabolic activity. Since viable cells contain NAD(P)H-dependent oxidoreductase enzymes that reduce the MTT reagent to formazan, cell survival was determined using the average of triplicate measurements taken at 570 nm using a plate reader from three independent experiments, as reported previously [28].

### 2.5. Western Blot Analysis

TLR2 and phosphorylated NF-κB proteins were isolated from the cytoplasmic extraction of Pam_3_CSK_4_-stimulated Raw264.7 cells using Proprep buffer, and subsequently detected using antibodies specific for TLR2 (1:2000, Cell Signaling Technology, Danvers, MA, USA), phosphorylated NF-κB (1:1000, Cell Signaling Technology, Danvers, MA, USA), and β-actin (1:5000, Santa Cruz Biotechnology, Dallas, TX, USA) as described previously [29]. Cultured cells were pretreated with 20 µM of CU-CPT22 and phloretin for 1 h, and then stimulated with 1 µg/mL Pam_3_CSK_4_. We measured the concentration of protein on a Nanodrop spectrophotometer (Thermo Scientific, Pittsburgh, PA, USA; range: 0.06–820 mg/mL bovine serum albumin). β-actin was used as the control in the Western blot. ImageJ software (National Institutes of Health, Bethesda, MD, USA) was used to quantify the relative intensities of the protein bands detected using these antibodies.

### 2.6. Binding Assay of TLR1, TLR2, and Phloretin

The binding between recombinant human TLR2 and TLR1 (R&D Systems, Minneapolis, MN, USA) and phloretin was determined using bio-layer interferometry (BLI; ForteBio, Menlo Park, CA, USA). A nickel–nitrilotriacetic acid sensor was used to analyze TLR binding. Biosensors were hydrated in phosphate-buffered saline for 10 min before the tests. The assay involved an initial 15-s baseline step, a 60-s loading step, a 30-s baseline step, a 55-s association step, and a 60-s dissociation step [18]. Recombinant human TLR2 and TLR1 (0.1 mg/mL each) were used to measure the binding affinities.

### 2.7. Molecular Docking Study

The molecular docking of phloretin was performed using Auto Dock [30] implemented in Yasara software. The crystal structure of the TLR2/1 (PDB ID: 2Z7X) heterodimer was used as a receptor after removing the water molecules and lipopeptide [31]. The entire protein dimer was set as the active site using the AutoGrid algorithm, and the “dock_run” protocol and Lamarck genetic algorithm were used for the docking procedure. The ligands were subjected to 100 docking runs, and the docking conformations were retrieved using the “dock_play” protocol.

### 2.8. Statistical Analysis

At least three independent cell samples were included in the statistical analysis using Graphpad Prism software. Dunnett’s multiple comparisons test (Prism 7.0, Graphpad Software Inc., La Jolla, CA, USA) was used for comparisons of multiple groups. Values were considered statistically significant at *p* < 0.05. The error bars represent ± standard error of measurement(SEM). * *p* < 0.05; ** *p* < 0.01; and *** *p* < 0.001 compared to cells treated with agonist. n.s. represents no significance.

## 3. Results

### 3.1. Phloretin Effectively Reduced the TNF-α Production through TLR2/1 Signaling in Raw264.7 Cells

**Figure 2:**
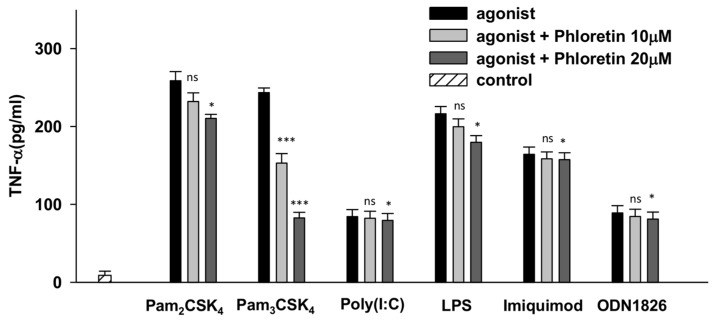
Specificity of phloretin with various TLR-specific agonists that selectively activate different TLRs determined by monitoring the inhibition activity of TNF-α production in Raw264.7 cells. Pam_3_CSK_4_ (200 ng/mL), Pam_2_CSK_4_ (200 ng/mL), poly(I:C) (1 µg/mL), LPS (20 ng/mL), imiquimod (1 µg/mL), and ODN1826 (10 µg/mL) were used to selectively activate respective TLRs. TNF-α secreted into the supernatant was measured by ELISA. Each sample was measured in triplicate. The error bars represent ± SEM. (* *p* < 0.05; *** *p* < 0.001). n.s. represents no significance, tumor necrosis factor (TNF), Toll-like receptors (TLRs), lipopolysaccharide (LPS), standard error of measurement (SEM).

Among the series of TLRs examined, phloretin was found to selectively and significantly inhibit TLR2/1 signaling in Raw264.7 cells by reducing 37.2% and 66.1% of the Pam_3_CSK_4_-induced TNF-α production at 10 µM and 20 µM, respectively. As shown in Figure 2, phloretin did not substantially inhibit TLR2/6 signaling in Pam_2_CSK_4_-stimulated Raw264.7 cells, with only a 10.3% and 18.7% reduction of TNF-α at 10 µM and 20 µM, respectively. Phloretin also only inhibited 7.7% and 16.9% of the LPS-induced TNF-α production (which activates TLR4 signaling) at 10 µM and 20 µM, respectively, in Raw264.7 cells. However, phloretin did not inhibit the TNF-α production induced by imiquimod, ODN1826, or poly (I:C). Therefore, phloretin most effectively reduced TNF-α production through TLR2/1 signaling.

### 3.2. Effects of Phloretin and CU-CPT22 on Proinflammatory Cytokines in Pam_3_CSK_4_-Stimulated HEK293-hTLR2 Cells

**Figure 3:**
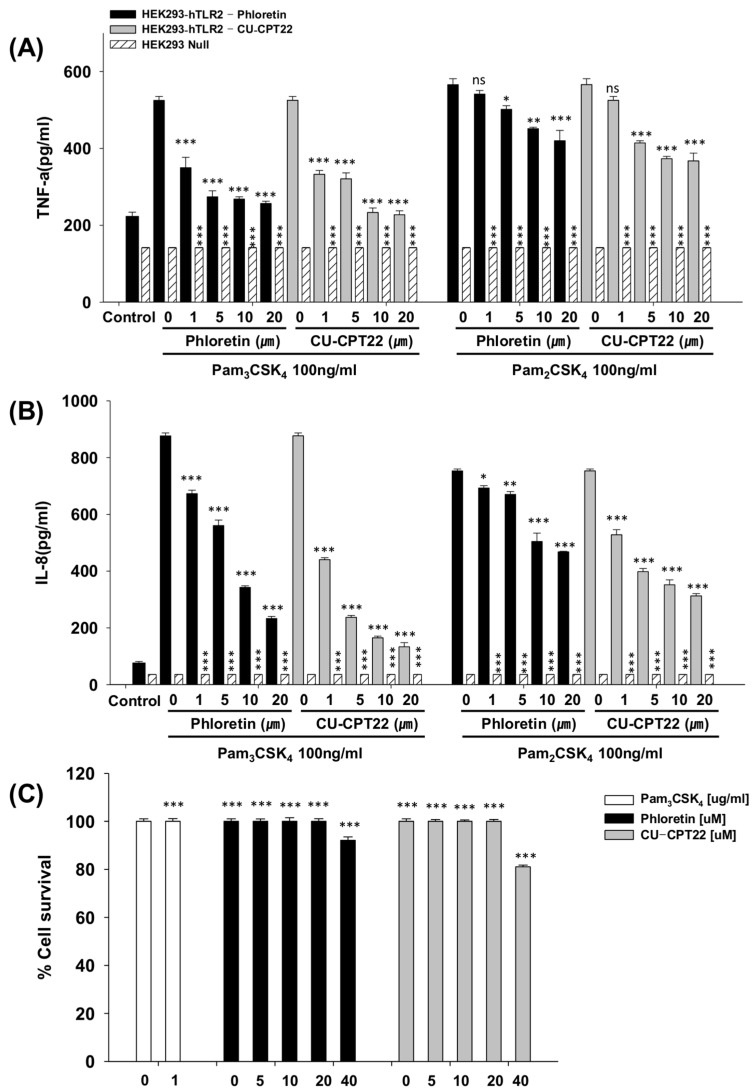
Effect of phloretin on cytokine levels, TNF-α (**A**) and IL-8 (**B**), in 100 ng/mL Pam_3_CSK_4_- and Pam_2_CSK_4_-stimulated HEK293-hTLR2 cells and HEK293-null cells. HEK293 cells were pretreated for 1 h with phloretin (1, 5, 10, 20 µM) or CU-CPT22 (1, 5, 10, 20 µM) before stimulation with the agonists, Pam_3_CSK_4_ or Pam_2_CSK_4_ (100 ng/mL), for 16 h. Supernatants were collected and the levels of TNF-α and IL-8 in Pam_3_CSK_4_- or Pam_2_CSK_4_-stimulated HEK293-hTLR2 cells were determined by ELISA. * *p* < 0.05, ** *p* < 0.01, and *** *p* < 0.001 compared to HEK293-hTLR2 cells treated with agonists only. (**C**) Concentration-dependent toxicity of phloretin, CU-CPT22, and Pam_3_CSK_4_ against HEK293-hTLR2 cells. *** *p* < 0.001 compared to HEK293-hTLR2 cells treated with agonists only. The error bars represent ± SEM. n.s. represents no significance.

We next investigated the inhibitory effect of phloretin on the secretion of inflammatory cytokines such as IL-8 and TNF-α in Pam_3_CSK_4_-activated HEK293-hTLR2 cells. As shown in Figure 3A, phloretin inhibited TNF-α production in a concentration-dependent manner by 33.3%, 47.8%, 48.9%, and 51.1% at 1, 5, 10, and 20 μM, respectively. In contrast, there was no TLR2-activated TNF-α production detected in HEK293-null cells. In addition, 1, 5, 10, and 20 μM of phloretin reduced IL-8 levels by 23.2%, 36.0%, 60.9%, and 73.4%, respectively, in Pam_3_CSK_4_-induced HEK293-hTLR2 cells. CU-CPT22 was identified as a TLR2/1 antagonist through small-molecule library screening, which is a benzotropolone molecule that effectively inhibits the Pam_3_CSK_4_-induced TLR2/1 heterodimerization in Raw264.7 cells [32]. Therefore, the inhibitory effects of phloretin were compared to those of the known inhibitor CU-CPT22 in Pam_3_CSK_4_-induced HEK293-hTLR2 cells to determine its potential effectiveness in clinical application. Treatment with 1, 5, 10, and 20 μM of CU-CPT22 decreased the TNF-α quantity by 36.7%, 38.9%, 55.6%, and 56.7%, and decreased the IL-8 level by 49.8%, 73.0%, 81.2%, and 84.8%, respectively.

Phloretin did not considerably change the levels of Pam_2_CSK_4_-induced TNF-α and IL-8 compared to those induced by Pam_3_CSK_4_, implying that phloretin does not significantly inhibit the heterodimerization of TLR2/6 compared to TLR2/1 heterodimerization.

### 3.3. Toxicity Against Raw264.7 Cells and HEK293-hTLR2 Cells

As shown in Figure 3C, the MTT assay confirmed that phloretin did not cause cytotoxicity against HEK293-hTLR2 cells at any concentration tested up to 20 µM. Incubation with 20 μM CU-CPT22 did not affect the survival rates of HEK293-hTLR2 cells (Figure 3C), implying that CU-CPT22 is not toxic against human HEK293-hTLR2 cells at the concentrations tested. At 40 μM, CU-CPT22 reduced the survival of HEK293-hTLR2 cells by 18.9%, while phloretin reduced survival by only 7.92%. Therefore, the present findings indicate that phloretin could be a viable substitute of CU-CPT22.

### 3.4. Effect of Phloretin and CU-CPT22 on Protein Expression in Pam_3_CSK_4_-Stimulated Raw 264.7 Cells

**Figure 4:**
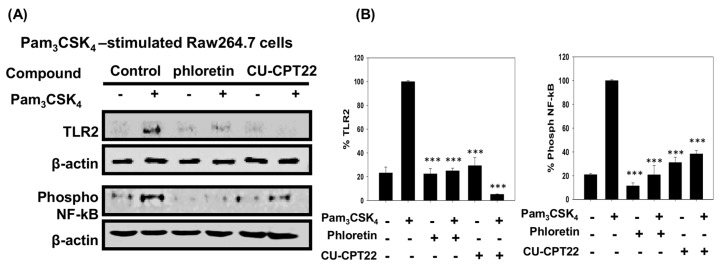
Quantification of protein expression using Western blotting. (**A**) Indicated protein levels of TLR2 and NF-κB phosphorylation were analyzed in Pam_3_CSK_4_-stimulated Raw 246.7 cells with (+) or without (−) phloretin or CU-CPT22. (**B**) Relative protein expression was quantified using Image J. *** *p* < 0.001 compared to HEK293-hTLR2 cells treated with Pam_3_CSK_4_ only. The error bars represent ± SEM.

As shown in Figure 4, the levels of TLR2 protein in Pam_3_CSK_4_-induced Raw 264.7 cells were suppressed by 75.1% and 94.9% in cells treated with 20 μM phloretin and 20 μM CU-CPT22 compared to controls, respectively. The extent of NF-κB phosphorylation, which regulates its nuclear translocation and activation of inflammatory cytokine gene transcription, was reduced by 79.2% and 61.7% in the presence of phloretin and CU-CPT22, respectively. These findings indicated that phloretin effectively inhibited TLR2/1 heterodimerization and reduced Pam_3_CSK_4_-induced inflammation by suppressing the level of TLR2 and proinflammatory cytokines, in a manner comparable to CU-CPT22.

### 3.5. Determination of Binding Affinity of Phloretin to TLR1 and TLR2 Using BLI

**Figure 5:**
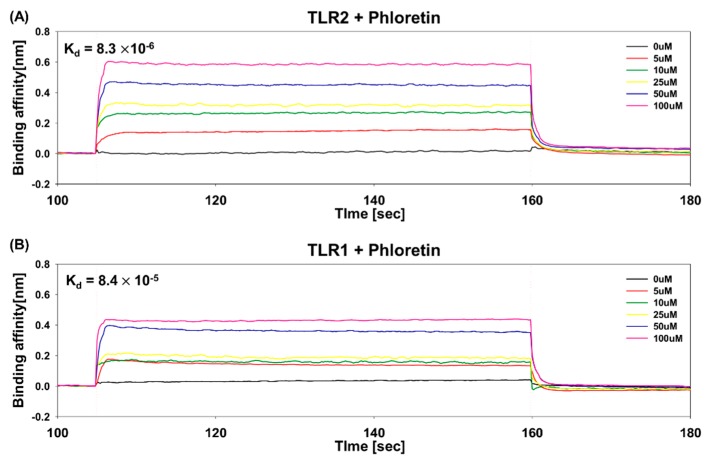
Binding of phloretin to TLR2 (**A**) and TLR1 (**B**) determined by bio-layer interferometry. Representative curves showing the phloretin association to and dissociation from immobilized TLR2 at various concentrations of phloretin (0, 5, 10, 25, 50, and 100 µM). Dissociation constant, K_d_ in M is marked in the figures.

The binding affinity of phloretin to TLR1 or TLR2 was measured using BLI, in which a change in the number of phloretin molecules bound to the TLR1 and TLR2 immobilized on the biosensor tip causes a wavelength shift in the interference pattern of TLRs. Binding of phloretin to TLR1 and TLR2 was monitored following the application of various concentrations (0, 5, 10, 25, 50, 100 μM) of phloretin. Only phloretin binding to or dissociating from the biosensor can shift the interference pattern, corresponding to the rates of association and dissociation. To determine binding parameters, data were analyzed using ForteBio Data Analysis Sofware 7.0.2.8. As shown in Figure 5, the binding affinity of phloretin to TLR2 was 8.3 × 10^−6^ M (association rate of 4.7 × 10^4^, dissociation rate of 3.9 × 10^−1^), while that to TLR1 was 8.4 × 10^−5^ M (association rate of 1.2 × 10^4^, dissociation rate of 1.0 × 10^−1^), indicating a high-affinity direct interaction. This binding affinity of phloretin to TLR2 and TLR1 determined using BLI indicated that phloretin might suppress TLR2 activation by directly binding to TLR2 and TLR1.

### 3.6. Docking Analysis of Phloretin with TLR2/1

**Figure 6:**
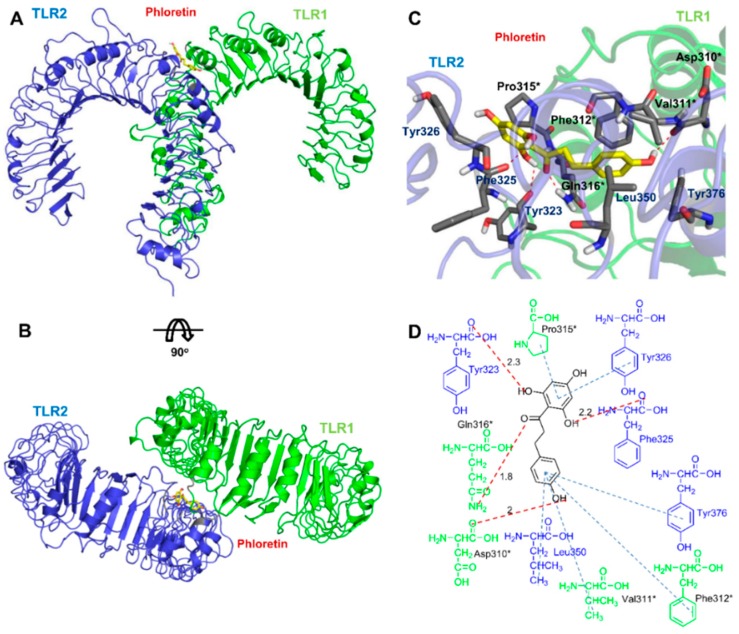
Model of phloretin binding to TLR2/1. (**A**) Overview of the TLR2/1–phloretin complex built using the X-ray structure of the TLR/1 complex (PDB ID: 2Z7X). TLR2, TLR1, and phloretin are displayed as a blue ribbon, green ribbon, and yellow stick, respectively. (**B**) Top view of phloretin in the predicted binding mode. (**C**) Active-site view of the docking pose. TLR2 and TLR1 residues participating in the interaction with phloretin are shown as gray sticks. Asterisks (*) denote TLR1 residues. Hydrogen bonds are shown as red dotted lines. (**D**) Two-dimensional depiction of the binding model. Hydrogen bonds and hydrophobic interactions are shown as red and blue dotted lines, respectively. Hydrogen bond distances (in angstroms) are shown above the dotted lines. TLR2 (blue) and TLR1 (green) residues participating in hydrogen bonding and hydrophobic interactions are shown.

Since the binding of phloretin to TLR2 and TLR1 was confirmed using the BLI assay, computational prediction of these interactions can contribute to the identification of the residues involved. To investigate the direct interaction between phloretin, TLR1, and TLR2, docking of phloretin to the TLR1–TLR2 interface was studied (Figure 6). The TLR2/1 complex structure (PDB ID: 2Z7X) without the lipopeptide was used to identify the binding mode of phloretin [31]. Since phloretin is much smaller than Pam_3_CSK_4_, it has less extensive interactions with the TLR1–TLR2-binding site compared to Pam_3_CSK_4_. In the predicted binding pose, similar interactions were detected that were observed in the TLR2/1 complex structure: the backbone oxygen atom of Phe325 in TLR2 forms a hydrogen bond with the 6′-OH in phloretin. Furthermore, a hydrogen bond was observed between the oxygen atom of phloretin and the side-chain nitrogen of Gln316 in TLR1. Similarly, the crystal structure of the TLR1 and triacyl lipopeptide complex revealed the presence of a hydrogen bond between the side-chain nitrogen of Gln316 of TLR1 and triacyl lipopeptide [31]. The phloretin A-ring has hydrophobic interactions with Tyr326 of TLR2 and Pro315 of TLR1. In addition, hydrophobic interactions were observed between the B-ring of phloretin and the Leu350 and Tyr376 residues of TLR2, as well as with the Val311 and Phe312 residues of TLR1. Distinctive hydrophobic interactions between the B-ring of phloretin and the aromatic ring of Tyr376 in TLR2 were also observed between phloretin and TLR2 that were not detected between Pam_3_CSK_4_ and the TLR2/TLR1 complex. In addition, the hydroxy group at the 4-position of the B-ring in phloretin forms a hydrogen bond interaction with the backbone oxygen atom of Asp310 in TLR1, and a hydrogen bond interaction was observed between the hydroxy group at the 2′-position of the A-ring in phloretin and the backbone oxygen in Tyr323 of TLR2. Pam_3_CSK_4_ has three acyl chains that mediate heterodimerization of the TLR2/1 complex: two lipid chains are inserted deep into TLR2 and one lipid chain is inserted into the hydrophobic channel of TLR1 [31]. Phloretin binds to the interface of TLR1 and TLR2 at a more shallow position compared to Pam_3_CSK_4_ [31]. This binding model confirmed the binding affinity data determined using BLI, indicating that phloretin inhibits TLR2–TLR1 heterodimerization by directly binding to TLR2 and TLR1.

## 4. Discussion

A growing number of natural compounds have been identified to show therapeutic benefits; thus, screening for such compounds and uncovering their mechanisms of action has becoming a focus of broad research. Phloretin is a versatile dietary natural compound that exhibits antioxidative and anti-inflammatory properties and induces apoptosis of several types of cancer cells. Here, we examined the TLR specificity of phloretin to uncover one of its key therapeutic properties toward development in the treatment of inflammatory diseases. We demonstrated that phloretin shows molecular interactions with TLR2/1 and modulates the TLR2 signaling pathway. The specificity for TLR2 was confirmed through suppression of TNF-α production induced mainly by the TLR2/1 agonist Pam_3_CSK_4_ among a series of TLR agonists tested in Raw264.7 cells. Moreover, phloretin effectively inhibited TLR2/1 heterodimerization and reduced Pam_3_CSK_4_-induced inflammation in human HEK293-hTLR2 cells by suppressing the level of proinflammatory cytokines with comparable effects to those of CU-CPT22. The potential TLR2 inhibition properties of phloretin were demonstrated by decreased expression levels of TLR2 and phosphorylation of NF-κB induced by Pam_3_CSK_4_ that activates the TLR2/1 heterodimerization, thereby inducing the production of proinflammatory cytokines. We further demonstrated the release of cytokines in the cell supernatant by ELISA. The mRNA expression levels of these cytokines in the cells can also be measured by RT-qPCR to provide more information about the effect on cytokine secretion.

Recently, several inhibitors of TLR2-induced proinflammatory cytokine production have been reported [32,33,34,35]. In addition to the well-known inhibitory molecule CU-CPT22 [16], virtual screening has been applied to find novel nonpeptide TLR2 antagonists [33] and small-molecule TLR2 antagonists with low-micromolar half-maximal inhibitory concentrations [34]. Flavonoids are secondary plant metabolites that are involved in the defense against environmental stress and pathogenic microbes. Zhong et al. [35] discovered an immunomodulatory natural-product-like compound, which was developed as a direct inhibitor of TLR2/1 heterodimerization. Although this compound has a different structure from that of phloretin, they share a similar predicted ligand-binding site at the TLR1 and TLR2 interface.

Despite these promising results, it is also important, but challenging, to identify the targets of small molecules that mediate their therapeutic effects. Since phloretin inhibited the Pam_3_CSK_4_-mediated stimulation of TLR2 in mouse Raw264.7 cells and human HEK293-hTLR2 cells, and phloretin binding to TLR2 and TLR1 was confirmed based on micromolar binding affinity using the BLI assay, we investigated the direct interaction between phloretin and TLR2 by docking calculation. Even though phloretin does not have lipid chains and is much smaller than Pam_3_CSK_4_, which has extensive interactions deep into the TLR2/1 complex, phloretin also showed efficient binding interactions to the TLR2 and TLR1 interface. Phloretin has two rings, which have a flexible linkage that may facilitate its binding to the TLR2 and TLR1 interface and efficient inhibition of TLR2 signaling. The molecular docking results showed that the hydrogen bonds between Pam_3_CSK_4_ and Phe327, as well as the Phe349 backbones of TLR2, in the Pam_3_CSK_4_–TLR2/1 crystal structure are conserved in the phloretin binding model [31]. Phloretin forms hydrophobic interactions efficiently between its rings and the hydrophobic residues at the TLR2–TLR1 interface. Furthermore, the hydroxyl groups of the A and B rings have extensive electrostatic interactions with TLR2 and TLR1. As seen in Figure 6, phloretin binds to a similar interaction site at the interface of TLR2 and TLR1 as observed for the binding of Pam_3_CSK_4_ to the TLR2/1 complex, indicating that phloretin may disrupt the heterodimerization of TLR2 with TLR1 by preventing the lipopeptide ligand from accessing the binding site. Furthermore, the phloretin–TLR2/1 binding model suggested that hydrophobic interactions between phloretin and Phe312 of TLR1 and the nearby residues may be critical for the specificity of phloretin to TLR2/1. Absence of the Phe residue in TLR6, which corresponds to the Phe312 residue of TLR1, may be the critical factor contributing to the higher preference of phloretin for the TLR2/1 complex compared to the TLR2/6 complex.

TLR2 plays important roles in the innate immune system and is related to many inflammatory diseases. Therefore, discovery of a TLR2 antagonist with low cytotoxicity such as phloretin shows great promise for the treatment of many immune-mediated inflammatory diseases. Further studies on its potential clinical application should help to elucidate the potency of phloretin on TLR2-related inflammation.

## 5. Conclusions

Since phloretin, a dihydrochalcone flavonoid, is abundant in apples and strawberries, it can form a common part of the daily diet. The present study demonstrated that phloretin can also be a potent phytocompound as an inhibitor of the heterodimerization of TLR2/1. We further uncovered the mechanism and target proteins related to the inhibitory effects of phloretin on TLR2 activation and related inflammatory signaling pathways. In particular, phloretin significantly inhibited TLR2/1 signaling and the upregulation of various cytokines, TLR2, and NF-κB phosphorylation induced by Pam_3_CSK_4_ in Raw 264.7 cells. Moreover, we showed that phloretin reduced the Pam_3_CSK_4_-induced inflammation in human HEK293-hTLR2 cells with comparable activity to that of CU-CPT22, mediated by the TLR2 pathway through direct interactions with TLR1 and TLR2. In conclusion, this study showed the great potential of phloretin to act as a potent natural TLR2/1 inhibitor and thereby suppress TLR2-induced inflammation.
